# Impact of recurrent IgA nephropathy on long-term kidney transplant outcomes: a cohort comparison with autosomal dominant polycystic kidney disease

**DOI:** 10.1186/s12882-026-05207-2

**Published:** 2026-08-13

**Authors:** Rashed Kamal, Mohamed Elewa, Mustafa Sevinc, Isabella Carpio-Gonzalez, Mohammed Meghji, Durga Kanigicherla

**Affiliations:** 1 The University of Manchester, Manchester Medical School Stopford Building, Oxford Road Manchester, Manchester, M13 9PT UK; 2 Manchester Institute of Nephrology & Transplantation, Manchester University NHS Foundation Trust, Oxford Road, Manchester, M13 9WL UK; 3 Faculty of Medicine, Ain Shams University, Cairo, Egypt; 4 Manchester University NHS Foundation Trust, Oxford Road, Manchester, M13 9WL UK

**Keywords:** Kidney transplantation, Recurrent IgA nephropathy, Graft survival, Autosomal dominant polycystic kidney disease, Disease recurrence

## Abstract

**Background:**

IgA nephropathy (IgAN) can recur following kidney transplantation and may compromise long-term graft survival. Autosomal dominant polycystic kidney disease (ADPKD) represents a non-recurrent comparator with excellent transplant outcomes. However, direct long-term comparisons between these diseases, particularly accounting for IgAN recurrence, remain limited.

**Methods:**

We retrospectively analysed kidney transplant recipients transplanted between 2010 and 2023 for IgAN (*n* = 137) or ADPKD (*n* = 122) as primary cause of end stage kidney disease at a large UK transplant centre. IgAN recipients were stratified by biopsy-confirmed recurrence. The primary outcome was death-censored graft survival (DCGS). Secondary outcomes included patient survival, causes of graft failure, and post-transplant renal function. Survival was assessed with Kaplan–Meier methods and Cox regression analyses.

**Results:**

Over a median follow-up of 6.38 years (IgAN) and 7.04 years (ADPKD), overall DCGS and recipient survival were comparable between the IgAN and ADPKD cohorts. However, recurrence identified a distinct high-risk subgroup within IgAN. Recurrence was observed in 12.4% of recipients and was associated with markedly inferior graft survival: 10-year DCGS was 35.8% in recurrent IgAN versus 88.8% in non-recurrent IgAN (*p* < 0.05). In univariate Cox regression, recurrence was associated with graft failure (HR 5.50, 95% CI 2.33–12.96). Patient survival did not differ by primary disease or recurrence status.

**Conclusion:**

Although overall long-term outcomes following transplantation are similar in IgAN and ADPKD, recurrence delineates a subgroup of IgAN recipients with substantially inferior graft survival. These findings highlight recurrent IgAN as a major driver of late allograft failure and underscore the need for sustained surveillance and strategies to mitigate recurrent disease.

**Supplementary Information:**

The online version contains supplementary material available at https://doi.org/10.1186/s12882-026-05207-2.

## Introduction

IgA nephropathy (IgAN) and Autosomal dominant polycystic kidney disease (ADPKD) are among the most common causes of end-stage kidney disease (ESKD) worldwide, yet they differ fundamentally in pathophysiology and post-transplant risk profiles [1, 2]. ADPKD is a non-immunological, structural kidney disease caused by heritable mutations in PKD genes, leading to progressive cyst growth, renal enlargement and functional impairment [3]. It accounts for approximately 10% of patients requiring renal replacement therapy [4]. In contrast, IgAN is an immune-mediated glomerulonephritis defined by mesangial IgA deposition, with a highly variable clinical course, ranging from asymptomatic haematuria to rapidly progressive kidney failure [5] It remains the most common cause of primary glomerular disease globally [1].

Kidney transplantation is the treatment of choice for both conditions; however, long-term outcomes differ. ADPKD recipients generally demonstrate excellent graft and recipient survival, although longer term risks of cardiovascular disease and malignancy remain [6, 7]. In contrast, outcomes following transplantation in IgAN recipients are more heterogenous. While many recipients achieve long-term graft survival comparable to other primary renal diseases, a substantial proportion experience progressive graft dysfunction [8]. A key contributor to this variability is the risk of recurrent IgAN in the allograft [9].

Reported recurrence rates vary widely, from 9% to over 60%, reflecting differences in biopsy practices, follow-up duration and definitions of recurrence [10]. Recurrence may be subclinical, detected on protocol biopsies, or clinically manifest with haematuria, proteinuria, and declining graft function [11]. Several factors including younger recipient age and HLA matching, have been proposed as risk factors, although findings remain inconsistent across studies [12]. Importantly, recurrent IgAN has been implicated as a cause of graft failure, with reported graft loss attributable to recurrence ranging from 2% to nearly 30% [13, 14].

Despite prior literature on recurrent IgAN following kidney transplantation, most studies reflect earlier immunosuppressive eras or employ heterogeneous comparator populations, limiting interpretation in contemporary clinical practice. Data evaluating the impact of recurrence under current tacrolimus-based, steroid-minimisation strategies remain limited, and few analyses have contrasted IgAN outcomes with a biologically distinct, non-recurrent kidney disease to contextualise long-term graft outcomes. These gaps are increasingly relevant as advances in IgAN pathobiology and emerging targeted therapies in the native kidney renew interest in defining clinically meaningful recurrence in the transplant setting. A clear understanding of the magnitude by which recurrence influences graft survival is essential not only for patient counselling and surveillance, but also for estimating the potential population-level benefit of interventions targeting immune-mediated disease in IgAN. Quantifying this clinical impact therefore provides a necessary framework for evaluating and designing future therapeutic strategies in transplant recipients.

Accordingly, we compared long-term transplant outcomes in recipients with IgAN and autosomal dominant polycystic kidney disease (ADPKD) at a large UK transplant centre, with additional stratification of IgAN recipients by recurrence status. ADPKD was selected as a biologically appropriate comparator, representing a non-recurrent structural kidney disease with favourable long-term outcomes, enabling clearer delineation of the contribution of disease recurrence to long-term allograft prognosis.

## Methods

### Study design and objectives

This was a single-centre, retrospective cohort study of kidney transplant recipients with primary kidney due to IgAN and ADPKD, undertaken to evaluate the impact of IgAN recurrence on long-term graft survival. The primary objectives were to (A) Identify the incidence of recurrence in IgAN and (B) Assess death-censored graft survival (DCGS) in IgAN in comparison to ADPKD. Secondary outcomes were patient survival, causes of graft loss, and identifying risk factors that influence survival in IgAN and ADPKD. Serial creatinine values were analysed as a marker of post-transplant graft function.

### Patient selection and data collection

We included consecutive adult kidney transplant recipients with IgAN and ADPKD who underwent transplantation at the Manchester Royal Infirmary between 2010 and 2023. In total, 137 patients received kidney transplants with IgAN as primary disease and 122 patients for ADPKD. The IgAN group was subdivided into non-recurrent IgAN (IgAN R-, *n* = 120) and recurrent IgAN (IgAN R+, *n* = 17). Primary diagnosis of IgAN was confirmed through biopsy of native kidneys and ADPKD was based on imaging studies (ultrasound or CT) or demonstration of genetic mutations from patient records. Patients with other causes of end stage kidney disease (ESKD) or multi-organ transplants were excluded. Follow-up extended from the date of transplantation until graft loss or patient death.

All recipients received centre-standard immunosuppression, including induction therapy with basiliximab. Maintenance immunosuppression followed a steroid minimisation protocol comprising tacrolimus and mycophenolate mofetil–based therapy, with corticosteroids routinely withdrawn after five days post-transplantation. This protocol was applied uniformly to both the IgAN and the ADPKD cohorts over the study period; no cohort received steroid-inclusive maintenance by design, and there were no disease-specific differences in the standard regimen.

Graft survival was defined as the time from transplantation to graft failure (requiring return to dialysis or re-transplantation). Deep phenotypic data was extracted from hospital and primary care medical records, including demographic information, clinical characteristics, donor graft characteristics, and HLA mismatch data. Indication biopsies were performed for suspected graft dysfunction (e.g., haematuria, proteinuria, or a decline in graft function). For IgAN patients, recurrence status was determined based on histopathological confirmation of recurrence on biopsy.

### Statistical analysis

Baseline characteristics were summarised using descriptive statistics, with continuous variables expressed as means (± SD) or medians (IQR) and categorical variables as frequencies. Group differences were assessed using Fisher’s exact tests for categorical variables and Wilcoxon rank-sum tests for continuous variables. Kaplan-Meier analysis estimated patient and death censored graft survival, with log-rank tests comparing curves. Cox proportional hazards models were fitted as univariate models. The cumulative incidence of recurrence was estimated with competing risk methods. Analysis was performed in R (version 4.5.3, using survival, cmprsk and lme4) figures were generated in R.

## Results

### Baseline characteristics

Baseline characteristics are summarised in Table 1. IgAN recipients were numerically more often male and were significantly younger than ADPKD recipients. Pre-transplant kidney replacement therapy (KRT) also differed significantly. Pre‑transplant KRT modality differed overall (*p* = 0.001), with ADPKD more often receiving pre‑emptive transplantation (35.2% vs. 18.2%) and IgAN recipients being more often on haemodialysis at the time or transplantation (62.0% vs. 47.5%). Donor type did not differ significantly between the groups.

**Table 1:** Baseline characteristics and clinical outcomes of ADPKD and IgA nephropathy kidney transplant recipients

Variables	All (*n* = 259)	ADPKD (*n* = 122)	IgAN (*n* = 137)	IgAN R- (*n* = 120)	IgAN R+ (*n* = 17)	ADPKD vs. IgAN *p*†
Demographics						
Male, n (%)	163 (62.9%)	63 (51.6%)	100 (73.0%)	86 (71.7%)	14 (82.4%)	< 0.001
Age at KT (years), mean (SD); median (range)	50.68 (12.28); 50.84 (20.15–76.76)	54.71 (10.20); 55.14 (20.86–76.76)	47.10 (12.89); 47.53 (20.15–76.64)	47.81 (13.09); 47.97 (20.15–76.64)	42.04 (10.40); 43.85 (20.65–60.82)	< 0.001
**Pre-transplant renal replacement therapy**						
No dialysis, n (%)	68 (26.3%)	43 (35.2%)	25 (18.2%)	24 (20.0%)	1 (5.9%)	0.001
Hemodialysis, n (%)	143 (55.2%)	58 (47.5%)	85 (62.0%)	72 (60.0%)	13 (76.5%)	0.001
Peritoneal dialysis, n (%)	48 (18.5%)	21 (17.2%)	27 (19.7%)	24 (20.0%)	3 (17.6%)	0.001
Prior KT, n (%)	34 (13.1%)	3 (2.5%)	31 (22.6%)	31 (25.8%)	0 (0.0%)	< 0.001
Time on dialysis before KT (years), mean (SD); median (range)	4.29 (4.98); 2.53 (0.00-23.10)	3.04 (2.56); 2.38 (0.00-14.91)	5.18 (5.99); 2.67 (0.07–23.10)	5.49 (6.29); 2.63 (0.25–23.10)	3.20 (2.99); 2.86 (0.07–10.90)	NS (0.263)
**Total HLA mismatch**,** mean (SD)**	2.90 (1.42)	2.93(1.38)	2.87(1.46)	2.87(1.48)	2.83(1.34)	NS
**Type of donor**						
Donation after brain death (DBD), n (%)	106 (40.9%)	47 (38.5%)	59 (43.1%)	54 (45.0%)	5 (29.4%)	NS (0.759)
Donation after circulatory death (DCD), n (%)	64 (24.7%)	32 (26.2%)	32 (23.4%)	27 (22.5%)	5 (29.4%)	NS (0.759)
Living donor transplant (LDT), n (%)	89 (34.4%)	43 (35.2%)	46 (33.6%)	39 (32.5%)	7 (41.2%)	NS (0.759)
**Kidney transplantation outcomes**						
Patient survival, 1 year (%)	NA	95.1	97.8	97.5	100.0	
Patient survival, 5 years (%)	NA	90.3	90.4	89.9	94.1	
Patient survival, 10 years (%)	NA	86.2	77.4	73.3	94.1	
Death-censored graft survival, 1 year (%)	NA	95.9	95.6	95.0	100.0	
Death-censored graft survival, 5 years (%)	NA	92.7	87.2	90.4	70.1	
Death-censored graft survival, 10 years (%)	NA	81.7	78.1	88.8	35.8	
Follow-up (years), mean (SD); median (range)	6.86 (3.83); 6.73 (0.22–14.35)	7.14 (3.93); 7.03 (0.22–14.35)	6.61 (3.74); 6.38 (0.47–14.33)	6.21 (3.68); 5.75 (0.47–14.33)	9.43 (2.95); 10.00 (4.11–13.14)	NS (0.265)
**Laboratory values**						
Creatinine at 3 months, median (IQR), umol/L	140 (62)	137 (52)	144 (62)	143 (65)	145 (52)	
Creatinine at 1 year, median (IQR), umol/L	128 (54)	122 (56)	134 (52)	128 (48)	157 (70)	
Creatinine at 5 years, median (IQR), umol/L	126 (75)	114 (58)	135 (80)	130 (73)	193 (69)	
Recurrence, n (%)	0 (0.0%)	0 (0.0%)	17 (12.4%)	0 (0.0%)	17 (100.0%)	
Graft loss, n (%)	38 (14.7%)	17 (13.9%)	21 (15.3%)	11 (9.2%)	10 (58.8%)	
**Causes of graft loss**						
Recurrent IgAN	7 (2.7%)	0 (0.0%)	7 (5.1%)	0 (0.0%)	7 (41.2%)	
IFTA	6 (2.3%)	4 (3.3%)	2 (1.5%)	2 (1.7%)	0 (0.0%)	
Rejection	12 (4.6%)	4 (3.3%)	8 (5.8%)	5 (4.2%)	3 (17.6%)	
Other / undetermined	13 (5.0%)	9 (7.4%)	4 (2.9%)	4 (3.3%)	0 (0.0%)	

**Abbreviations**: ADPKD, autosomal dominant polycystic kidney disease; IgAN, IgA nephropathy; IgAN R-, non-recurrent IgA nephropathy; IgAN R+, recurrent IgA nephropathy; KT, kidney transplantation; RRT, renal replacement therapy; ESKD, end-stage kidney disease; DBD, donation after brain death; DCD, donation after circulatory death; LDT, live donor transplant; HLA, human leukocyte antigen; SD, standard deviation; IQR, interquartile range; IFTA, interstitial fibrosis and tubular atrophy; TCMR, T-cell mediated rejection; NS, non-significant

**Notes**: Data are expressed as n (%), mean (± SD), or median (IQR/range) as indicated

† Statistical significance (*p* < 0.05) comparing the ADPKD (*n* = 122) and IgAN (*n* = 137) cohorts. Post-transplant renal function reported as serum creatinine in µmol/L Death-censored graft survival calculated excluding deaths with a functioning graft

### Recurrent disease (IgAN R + and IgAN R-)

Among 137 IgAN recipients, 17 recipients (12.4%) developed biopsy-confirmed recurrence (IgAN-R+). Baseline characteristics were broadly similar between the IgAN-R + and IgAN-R− groups. Mean total HLA mismatch did not differ significantly between groups (2.83 ± 1.34 vs. 2.87 ± 1.48, *p* = 0.54). Follow-up was longer in the IgAN R+ group compared with the R− group (10 vs. 5.8 years).

### Graft survival

**Fig. 1:**
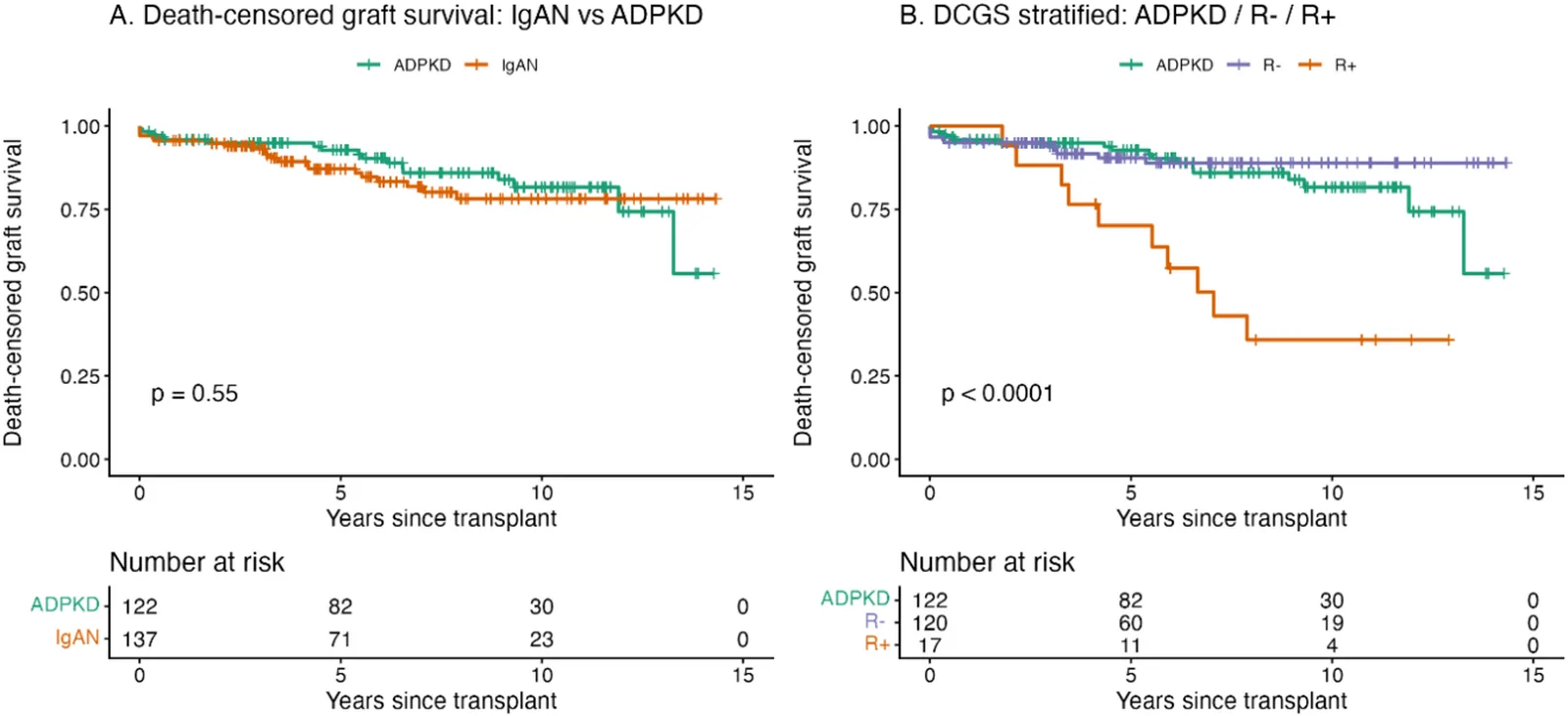
Death-censored graft survival. Kaplan-Meier analysis of graft survival in (**A**) ADPKD vs. IgAN recipients and (**B**) ADPKD vs. IgAN stratified by recurrence status (R + vs. R-). The log-rank test was used for comparisons

Death censored graft survival (DCGS) was comparable between IgAN and ADPKD recipients (Fig. 1A). 1-year DGCS was 95.6% IgAN vs. 95.9% ADPKD, 5-year 87.2% in IgAN vs. 92.7% in ADPKD. At 10 years, DCGS was 78.1% in IgAN and 81.7% in ADPKD recipients.

### Graft outcomes and effect of recurrence

Within the IgAN cohort, graft survival differed markedly according to recurrence status (Fig. 1B). 10-year DCGS was significantly lower in IgAN R+ recipients (35.8%) compared with IgAN R- recipients (88.8%) Fig. 1B extends this comparative analysis of IgAN and ADPKD by stratifying the IgAN group into recurrent (R+) and non-recurrent (R-) subgroups. Univariate Cox regression analysis demonstrated that recurrent IgAN was strongly associated with graft failure (HR 5.50, 95% CI 2.33–12.96, *p* < 0.001; Table 2). Other pre-transplant variables, including age, sex, donor type, and RRT modality, were not significantly associated with graft survival in IgAN recipients. In the ADPKD cohort, donation after circulatory death (DCD) was associated with an increased hazard of graft loss (HR 7.26, 95% CI 1.54–34.33, *p* = 0.012), while no other variables showed a significant association.

**Table 2:** Univariate Cox regression analysis of pre-transplant factors associated with death-censored graft failure

Variable	ADPKD HR (95% CI)	*p* value	IgAN HR (95% CI)	*p* value
Sex: male vs. female	2.83 (0.91–8.78)	NS	1.5 (0.51–4.47)	NS
Age at transplant, per year	1.01 (0.96–1.06)	NS	0.97 (0.94–1.01)	NS
Donor: DBD vs. living donor	3.34 (0.69–16.15)	NS	1.31 (0.5–3.45)	NS
Donor: DCD vs. living donor	7.26 (1.54–34.33)	0.012	0.89 (0.26–3.05)	NS
Pre-emptive transplant vs. dialysis	0.51 (0.16–1.56)	NS	0 (0-Inf)	NS
Dialysis vintage, per year	1.08 (0.86–1.34)	NS	0.95 (0.86–1.03)	NS
Prior kidney transplant	5.62 (0.7-44.77)	NS	0.57 (0.17–1.92)	NS
IgAN recurrence: R + vs. R-	--	--	5.5 (2.33–12.96)	< 0.001

**Abbreviations**: ADPKD, autosomal dominant polycystic kidney disease; IgAN, IgA nephropathy; HR, hazard ratio; CI, confidence interval; LDT, live donor transplant; DBD, donation after brain death; DCD, donation after circulatory death; RRT, renal replacement therapy; NS, non-significant

We estimated the cumulative incidence of recurrence, accounting for the competing risks of death and graft loss. Cumulative incidence rose progressively over time, from 2.2% at 1 year to 6.8% at 5 years and 14.6% at 10 years, with a median time from transplantation to recurrence of 60.6 months (range 7-132; Supplementary Figure S1).

The recurrent IgAN cohort was characterised further to explore whether the excess graft loss was concentrated in a subgroup with aggressive disease (Supplementary Table S1). Graft loss occurred in 10 of 17 recipients (59%), while 7 retained graft function at last follow-up. When recurrent patients were stratified by graft outcome as a proxy for aggressive versus indolent disease, neither the time to recurrence (median 54 vs. 86 months; *p* = 0.42) nor the one-year serum creatinine (172 vs. 157 µmol/L; *p* = 0.60) differed significantly between those who lost the graft and those who retained function; given the small number of events, these comparisons are under-powered and are reported descriptively rather than as evidence of a distinct prognostic subgroup. In a mixed-effects model anchored to the time of recurrence, serum creatinine rose by 16.3 µmol/L per year following recurrence (*p* = 0.001), consistent with accelerated functional decline after clinically manifest disease.

### Causes of graft loss

**Fig. 2:**
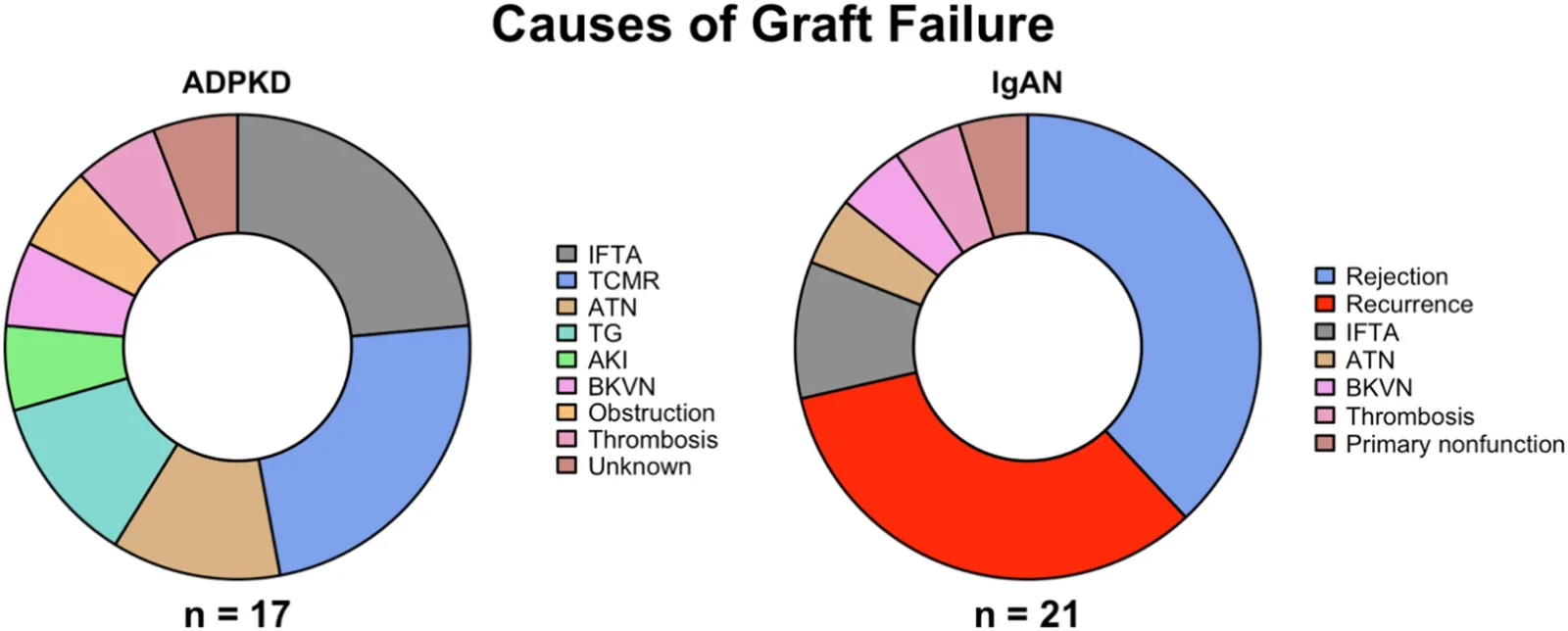
Causes of graft failure. Pie charts showing the distribution of causes for death-censored graft failure in ADPKD (*n* = 17) and IgAN (*n* = 21) cohorts. Categories are recurrent IgA nephropathy, T cell mediated rejection (TCMR), interstitial fibrosis and tubular atrophy (IFTA), and other or undetermined causes: Acute tubular necrosis (ATN), BK virus nephropathy (BKVN)

Causes of graft failure are shown in Fig. 2. In IgAN recipients, graft loss was most commonly attributed to rejection and recurrent disease. In contrast, graft failure in ADPKD recipients was primarily due to rejection and chronic interstitial fibrosis with tubular atrophy (IFTA). Multivariable modelling was not undertaken due to the limited number of graft failure events, which would have resulted in insufficient statistical power and a high risk of overfitting.

### Post-transplant kidney function

**Fig. 3:**
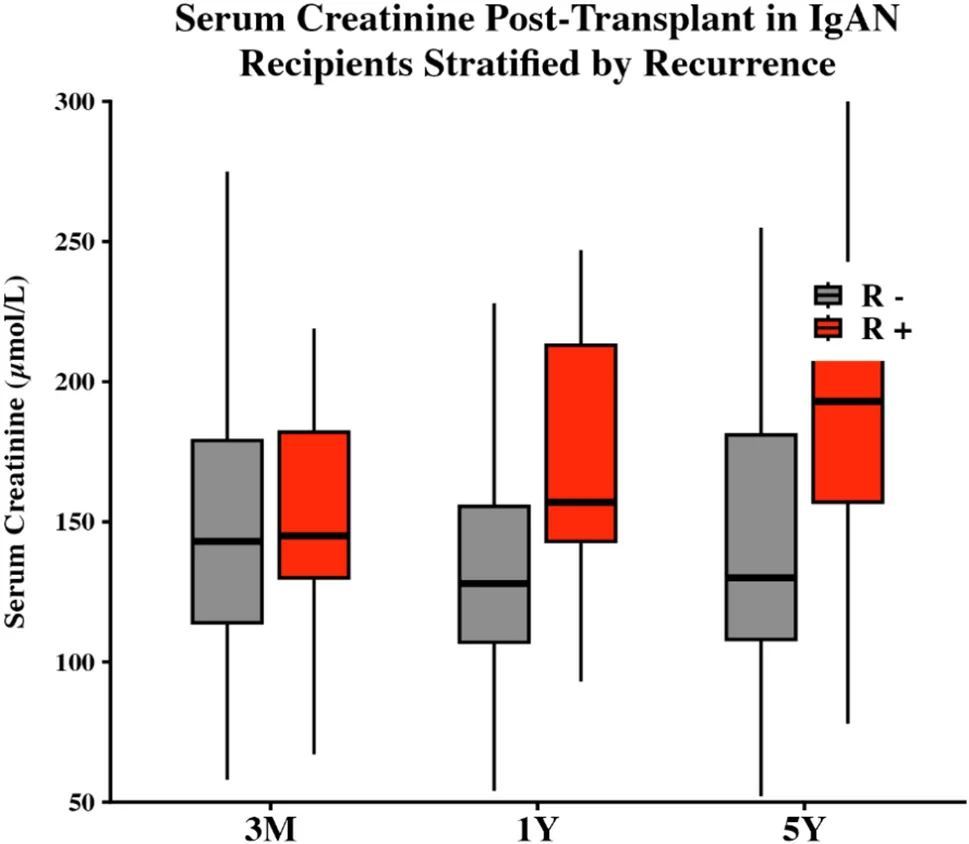
Post-transplant serum creatinine in IgAN recipients. Boxplot of serum creatinine at 3 months, 1 year, and 5 years post-transplant, comparing IgAN patients with recurrence (R+) and without (R-). Boxes show interquartile range (IQR) and median

Post-transplant kidney function was impaired in the IgAN R+ group. Median creatinine levels were consistently higher in IgAN-R+ compared to IgAN-R- group at 3 months (145 vs. 143), 1 year (157 vs. 128) and 5 years (193 vs. 130) (Fig. 3).

**Fig. 4:**
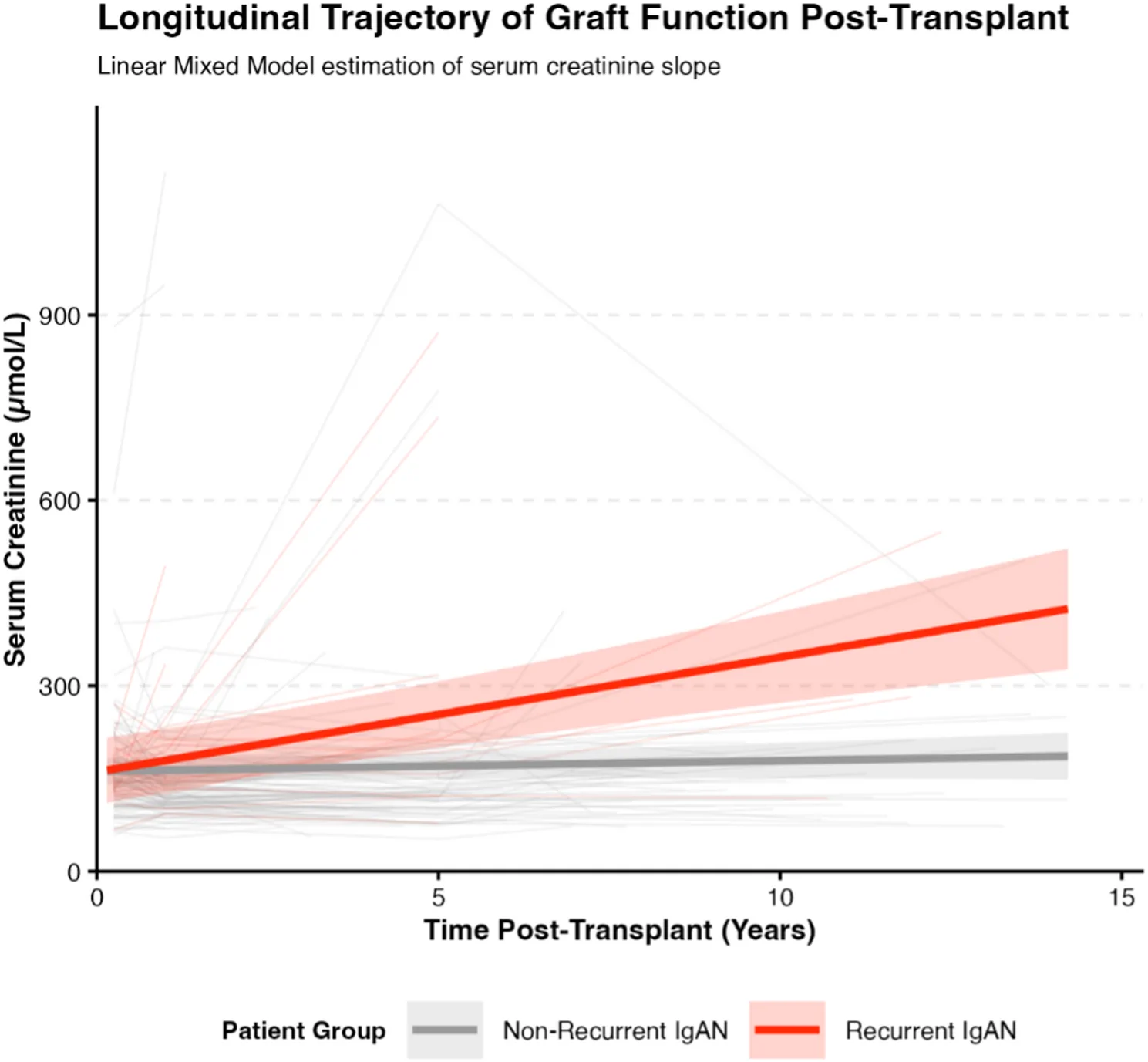
Longitudinal post-transplant serum creatinine trajectory in IgAN recipients stratified by recurrence status. Serial serum creatinine measurements are plotted over time after transplantation for recurrent and non-recurrent IgAN recipients. Individual patient trajectories are shown as faint lines, with modelled group-level trends and 95% confidence interval shading shown for each recurrence group. Recurrent IgAN was associated with a steeper rise in serum creatinine over follow-up compared with non-recurrent IgAN, consistent with progressive graft dysfunction in the recurrent subgroup

Conventional cross-sectional comparisons of renal function at fixed timepoints are inherently susceptible to survivor bias, as graft failures are excluded from later assessments, potentially overestimating stability within the cohort. To more accurately characterise longitudinal graft function and incorporate all available follow-up data, we performed a linear mixed-effects analysis to model the rate of functional change over time. Although early post-transplant renal function did not differ between recurrent and non-recurrent groups (*p* = 0.84), their trajectories diverged significantly. The non-recurrent group demonstrated a stable course (slope + 2.2 µmol/L/year, *p* = 0.18), whereas recurrent IgAN was associated with progressive functional decline, with an additional increase in serum creatinine of 16.3 µmol/L per year compared with non-recurrent cases (*p* < 0.001) (Fig. 4).

### Recipient survival

**Fig. 5:**
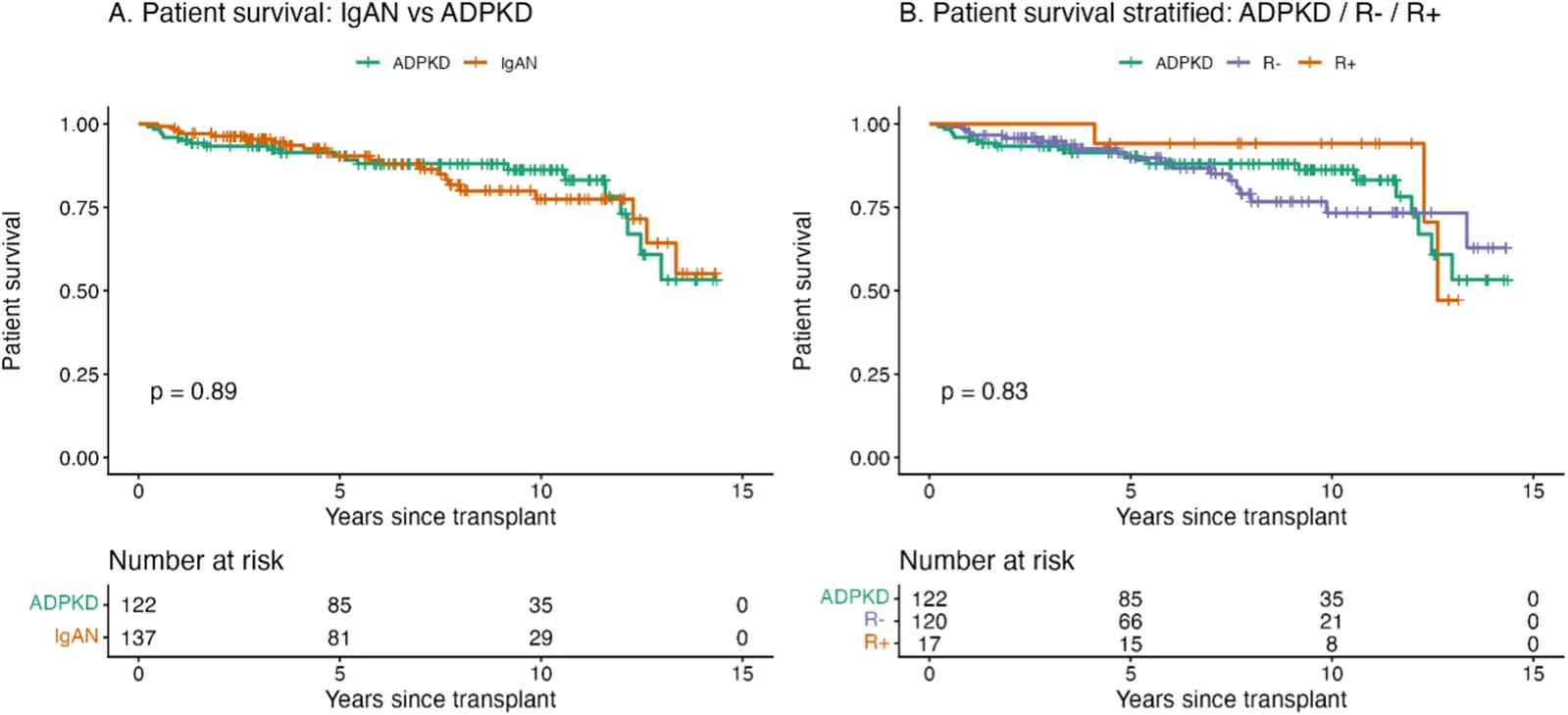
Post-transplant patient survival. Kaplan-Meier analysis comparing patient survival in (**A**) ADPKD vs. IgAN cohorts and (**B**) ADPKD vs. IgAN stratified by recurrence (R + vs. R-). Comparisons were made using the log-rank test

Recipient survival was excellent and comparable between IgAN and ADPKD recipients at 1 and 5 years (97.8% vs. 95.1% at 1 year; 90.4% vs. 90.3% at 5 years). At 10 years, patient survival was numerically lower in IgAN recipients (77.4%) compared with ADPKD recipients (86.2%), although this difference was not statistically significant (Fig. 5A).

Within the IgAN cohort, recipient survival did not differ significantly according to recurrence status (Fig. 5B). One-, five-, and ten-year survival rates were similar between IgAN R + and R- recipients, indicating that recurrence predominantly affected graft outcomes rather than patient mortality.

## Discussion

In this single-centre retrospective cohort study from one of the largest transplant centres in the UK, we compared long-term transplant outcomes in recipients with IgA nephropathy (IgAN) and autosomal dominant polycystic kidney disease (ADPKD), with specific evaluation of the impact of recurrent IgAN on allograft survival. Overall patient and graft survival were comparable between IgAN and ADPKD recipients during the first five years following transplantation. However, divergence emerged at longer follow-up within the IgAN cohort, driven by disease recurrence. This pattern aligns with previous reports demonstrating favourable short- to medium-term outcomes in IgAN, followed by deterioration in long-term graft survival, whereas ADPKD recipients maintain among the most favourable long-term post-transplant outcomes [15–17].

Recurrent IgAN was associated with a more than fivefold increased hazard of graft failure and a profound reduction in 10-year death-censored graft survival compared with non-recurrent IgAN. These findings reinforce recurrent IgAN as a major determinant of late graft loss rather than a benign histological finding. The magnitude of effect observed is consistent with prior cohort studies, including the TANGO study, which reported a 3.7-fold increased risk of graft failure associated with recurrence, and other series identifying recurrence as a leading contributor to late allograft loss [13, 18, 19]. Importantly, recurrence in our cohort was defined by clinically indicated biopsy, suggesting that the observed effect reflects disease with direct functional consequences rather than subclinical histological recurrence alone.

The demographic profile of our cohort reflect established epidemiology. IgAN recipients were younger at transplantation than ADPKD recipients [20, 21]. and demonstrated a male predominance, in keeping with registry and single-centre studies [13, 15, 20]. Despite younger age, IgAN recipients were less likely to receive pre-emptive transplantation and experience longer dialysis exposure prior to transplant likely reflecting the less predictable trajectory of kidney function decline compared with ADPKD [16, 17]. Given the established benefits of pre-emptive transplantation, this disparity merits further investigation.

Within the IgAN cohort, recurrence defined two distinct prognostic trajectories. Baseline recipient, donor, and transplant characteristics, including HLA mismatch, were broadly similar between recurrent and non-recurrent groups, and no strong pre-transplant predictors of recurrence were identified. In the absence of reliable risk stratification tools, these findings emphasise the importance of sustained clinical surveillance and timely diagnosis of recurrence to mitigate its impact on graft longevity.

Notably, recurrent IgAN was not associated with increased patient mortality. This dissociation between graft and patient outcomes observed in other studies likely reflects the availability of dialysis and re-transplantation following graft failure [12, 13, 15], Patterns of graft failure differed between the two diseases. In ADPKD, graft loss was predominantly attributed to rejection and chronic interstitial fibrosis with tubular atrophy, whereas in IgAN, recurrent disease accounted for a substantial proportion of failures in addition to rejection, reflecting the combined burden of alloimmune and disease-specific immune injury.

Although our findings corroborate existing literature, this study provides important contemporary context. Outcomes were evaluated in a modern transplant era characterised by tacrolimus-based immunosuppression and early steroid withdrawal, allowing reassessment of recurrence-associated risk under current practice patterns. The use of ADPKD as a biologically distinct, non-recurrent comparator offers a stable clinical reference, enabling clearer attribution of late graft divergence specifically to disease recurrence. By quantifying the magnitude by which recurrence drives long-term outcome divergence, these data also help define the potential population-level benefit of immune-targeted interventions in transplant recipients with IgAN.

From a clinical perspective, these findings highlight the importance of disease-specific counselling and sustained post-transplant surveillance in IgAN recipients. While early outcomes are favourable, recurrent disease may substantially compromise long-term graft survival. Accordingly, emergence of proteinuria, haematuria, or declining graft function should prompt timely investigation and consideration of biopsy.

### Limitations

This study has several limitations. Its single‑centre, retrospective design and the modest number of graft‑failure events limit generalisability, preclude multivariable adjustment, and contributed to the wide confidence intervals for recurrence estimates with potential for residual confounding. Because biopsies were performed for clinical indication rather than by protocol, only clinically manifest recurrence was captured, and subclinical disease may be under‑ascertained. Donor‑specific antibodies, microscopic haematuria, SGLT2 inhibitor use, and post‑recurrence treatment modifications were not systematically recorded. These limitations are offset by extended follow‑up at a high‑volume centre, uniform contemporary immunosuppression, and the use of ADPKD as a biologically non‑recurrent comparator. Several questions remain beyond the scope of this retrospective analysis. Whether earlier identification and intervention at the time of clinically manifest recurrence can alter the subsequent course of graft function is unknown, as is whether therapies targeting alloimmune injury benefit disease‑specific recurrent injury, or whether emerging disease‑modifying treatments for native IgAN will translate into improved allograft outcomes. Addressing these questions will require prospective, protocol‑biopsy studies with systematic capture of proteinuria, immunosuppression changes, and novel therapies.

## Conclusion

Kidney transplant outcomes in IgA nephropathy and autosomal dominant polycystic kidney disease are broadly comparable at the overall cohort level; however, recurrence of IgAN identifies a distinct high-risk subgroup with substantially inferior long-term graft survival. Although observed in a minority of recipients, recurrence was associated with a fivefold increased risk of graft loss and markedly reduced 10-year graft survival compared with non-recurrent IgAN and with outcomes observed in the non-recurrent comparator disease, ADPKD. These findings underscore the importance of sustained post-transplant surveillance and provide a clinical framework for evaluating emerging disease-targeted strategies aimed at preserving allograft function.

## Supplementary Information

Below is the link to the electronic supplementary material.

Supplementary Material 1

## Data Availability

The datasets analysed during the current study are not publicly available due to the risk of participant re-identification in the context of a single-centre transplant cohort but are available from the corresponding author on reasonable request.

## Abbreviations

IgAN: IgA nephropathy
ADPKD: Autosomal dominant polycystic kidney disease
IgAN R+: Recurrent IgA nephropathy
IgAN R−: Non-recurrent IgA nephropathy
ESKD: End-stage kidney disease
KT: Kidney transplantation
DCGS: Death-censored graft survival
RRT: Renal replacement therapy
HLA: Human leukocyte antigen
DBD: Donation after brain death
DCD: Donation after circulatory death
LDKT: Living donor kidney transplantation
IFTA: Interstitial fibrosis and tubular atrophy
TCMR: T-cell–mediated rejection
HR: Hazard ratio
CI: Confidence interval
SD: Standard deviation
IQR: Interquartile range
