# Supplementary material for: Impact of recurrent IgA nephropathy on long-term kidney transplant outcomes: a cohort comparison with autosomal dominant polycystic kidney disease
Source: BMC Nephrol. 2026 Aug 13;27:665. doi: 10.1186/s12882-026-05207-2 (PMC13753379; doi:10.1186/s12882-026-05207-2)
Supplement: Supplementary file 1 [file 12882_2026_5207_MOESM1_ESM.docx]

**Supplementary Table S1. Characterisation of the recurrent IgA nephropathy (IgAN R+) cohort, overall and stratified by graft outcome.**

**A. Summary by graft outcome**

| **Group** | **n** | **Time to recurrence, months, median (IQR)** | **Median age at Tx, years** | **Y1 creatinine, median, µmol/L** | **Follow-up, years, median** |
| --- | --- | --- | --- | --- | --- |
| All R+ | 17 | 60.6 (34.8–86.2) | 43.9 | 157 | 6.0 |
| Graft lost | 10 | 53.8 (35.9–64.4) | 45.3 | 172 | 4.9 |
| Function preserved | 7 | 86.2 (35.5–123.4) | 37.8 | 157 | 10.7 |

**B. Individual recurrent-IgAN recipients (n = 17)**

| **Outcome** | **Donor type** | **Time to recurrence (mo)** | **Age at Tx (yr)** | **Y1 creatinine (µmol/L)** | **Follow-up (yr)** |
| --- | --- | --- | --- | --- | --- |
| Graft lost | Living (LDT) | 65.3 | 32.8 | 135 | 5.5 |
| Graft lost | DBD | 8.9 | 47.5 | 247 | 1.8 |
| Function preserved | DCD | 132.2 | 51.4 | 213 | 12.9 |
| Graft lost | DBD | 92.3 | 46.7 | 234 | 7.9 |
| Function preserved | DCD | 122.5 | 36.8 | 167 | 12.0 |
| Graft lost | Living (LDT) | 39.3 | 48.4 | 124 | 3.3 |
| Function preserved | Living (LDT) | 38.8 | 48.0 | 157 | 11.1 |
| Function preserved | DBD | 124.3 | 36.7 | 101 | 10.7 |
| Graft lost | Living (LDT) | 67.8 | 35.4 | 93 | 6.7 |
| Graft lost | Living (LDT) | 47.0 | 48.4 | 148 | 4.2 |
| Function preserved | DBD | 86.2 | 60.8 | 143 | 8.1 |
| Graft lost | Living (LDT) | 60.6 | 26.2 | 193 | 5.9 |
| Graft lost | Living (LDT) | 61.9 | 55.7 | 151 | 7.1 |
| Function preserved | DCD | 7.2 | 37.8 | 205 | 4.1 |
| Graft lost | DCD | 34.8 | 43.9 | 335 | 3.4 |
| Function preserved | DCD | 32.3 | 37.5 | 146 | 6.0 |
| Graft lost | DBD | 8.5 | 20.7 | 494 | 2.1 |

Stratification by graft outcome (graft lost vs. function preserved) is used as a proxy for aggressive versus indolent recurrence. Differences between outcome groups were not statistically significant for time to recurrence (median 53.8 vs. 86.2 months; Wilcoxon p = 0.42) or one-year serum creatinine (172 vs. 157 µmol/L; p = 0.60); with only 17 patients and 10 events these comparisons are under-powered and are presented descriptively. In a mixed-effects model anchored to the time of recurrence, serum creatinine increased by approximately 16.3 µmol/L per year following recurrence (p < 0.001).
